# Effect of zearalenone in sugar beet products on zootechnical and reproductive performance and lesions of sows and piglets

**DOI:** 10.1007/s12550-024-00564-z

**Published:** 2024-10-12

**Authors:** A. Grümpel-Schlüter, S. Kersten, J. Kluess, S. Lühken, J. Saltzmann, A. Schubbert, S. Büngener-Schröder, S. Dänicke

**Affiliations:** 1 Institute of Animal Nutrition, Friedrich-Loeffler-Institut, Bundesallee 37, 38116 Brunswick, Germany; 2 Institute of Animal Welfare and Animal Husbandry, Friedrich-Loeffler-Institut, Dörnbergstraße 25/27, 29223 Celle, Germany; 3 GS Agri eG, Raiffeisenstraße 4, 49685 Schneiderkrug, Germany

**Keywords:** Sows, Piglets, Mycotoxins, Zearalenone, Reproductive performance, Lesions

## Abstract

Following the use of sugar beet pulp that was retrospectively found to be predominantly contaminated with zearalenone (ZEN) in diets of reproducing sows largely exceeding the EU-guidance value for critical ZEN concentration of 0.25 mg/kg, farmers did not report any changes in the reproductive performance of sows. Thus, the aim of the study was to verify this guidance value in a dose–response setup by using sugar beet pulp as a ZEN source hitherto not considered a risky feedstuff additionally characterized by comparatively low levels of deoxynivalenol. A total of 90 sows was equally allocated to one of the three feed groups during experimental lactation 1 and up to 40 days after insemination: CON with a minimal ZEN concentration, ZEN1 with a target concentration of 250 µg ZEN/kg feed, and ZEN2 with a target concentration of 500 µg ZEN/kg feed. Thereafter, all sows received the same feed without ZEN for the rest of gestation, and the following lactation for testing of putative carry-over effects resulting from previous ZEN exposure. Exposure of sows to ZEN with blood serum as an indicator was linearly related to dietary ZEN concentrations. Reproductive and zootechnical performances of sows were only affected by ZEN exposure at weaning weight. Clinical-chemical parameters indicated no clear effect of ZEN exposure. An influence of ZEN on the occurrence of tail and ear injuries (not necrosis) in piglets and lesions on the mammary complexes in sows is possible. The influence of a ZEN concentration above the EU guidance value on the study farm can therefore not be neglected.

**Supplementary Information:**

The online version contains supplementary material available at 10.1007/s12550-024-00564-z.

## Introduction

The contamination of feed with mycotoxins is a permanent challenge in animal nutrition, as these can have an impact on the performance and health of the animals as well as on the safety of the animal products (Döll and Dänicke 2011). Besides a number of commonly occurring mycotoxins such as aflatoxins, deoxynivalenol (DON), T-2 toxin, fumonisins, and ochratoxin A, zearalenone (ZEN) is of great importance because of its estrogenic properties (Murugesan et al. 2015; Santos Pereira et al. 2019). ZEN belongs to the group of *Fusarium*-toxins (Kanora and Maes 2009). In pigs, ZEN is metabolized after ingestion, and the predominant metabolite is α-zearalenol (Malekinejad et al. 2006), which has a higher toxicity than ZEN (Tiemann and Dänicke 2007). ZEN and its metabolites act at the estrogenic pre-receptor level and at estrogen receptors due to their structure mimicking that of estrogens. Thus, they interfere with estrogen metabolism and compete with the body’s own estrogens at the binding sites of estrogen receptors (Döll and Dänicke 2011; Minervini and Aquila 2008; Tiemann and Dänicke 2007). Symptoms such as hyperestrogenism, anoestrus, pseudopregnancy, and reduced reproductive performance can be caused by ZEN exposure in pigs (Binder et al. 2017; Kanora and Maes 2009). Especially during insemination and early pregnancy, it can have negative effects such as increased insemination costs due to more frequent insemination attempts or early embryonic death (EFSA Panel on Contaminants in the Food Chain 2011). Furthermore, ZEN can cause externally visible lesions in female pigs, e.g., swelling and reddening of the vulva and the mammary complexes (Döll et al. 2003; Yu and Pedroso 2023; Zhou et al. 2022). In addition to the effects concerning the sow, ZEN can also be transmitted to piglets via placenta (Dänicke et al. 2007; Goyarts et al. 2010) or milk (Palyusik et al. 1980), which can lead to an accumulation of mycotoxins in piglets’ blood (Goyarts et al. 2010), or lesions in the urogenital tract, the mammary gland or on claws, tails, and ears (Dacasto et al. 1995; Weissenbacher-Lang et al. 2012). In addition, there are indications that such effects might occur also sometime after direct oral exposure: previous toxicokinetic studies with radioactively labeled ZEN reported an accumulation in adipose tissue in rodents and laying hens after single oral administration (Ueno et al. 1977; Dailey et al. 1980). Whether toxins are indeed accumulated over a certain time span in body fat and thus might be released into the circulatory system during the break-down of such body reserves, i.e., during lactation, even well after the actual oral ZEN exposure of sows, is an ongoing debate for practitioners for years. Thus, experimental evidence for such putative carry-over effects is imperative.

As the effects caused by ZEN vary both in different livestock species and depending on age and direction of use, the European Commission has defined specific maximum tolerable ZEN concentrations as guidance values based on the results of recent studies (European Commission 2016). For gilts, the guidance value is 100 µg ZEN/kg feed (compound diet with 88% dry matter content), and for cyclic sows, it is 250 µg ZEN/kg feed (European Commission 2016).

Commercial pig farms in Germany reported the use of ZEN-contaminated molassed sugar beet pulp, originating from the 2018/19 beet campaign, in breeding sow diets, exceeding the guidance value for sow diets up to two- or threefold (Dänicke et al. 2024). According to the farm reports, no effects on the reproductive performance of sows could be determined, nor were their piglets affected by the increased ZEN levels in the sow feed. Subsequent laboratory analyses of sugar beet pulp batches showed that it was only slightly contaminated with DON in comparison to a high ZEN load of up to 10 mg/kg. Since most studies investigate the exposure to both mycotoxins, as they are often co-contaminants in feedstuffs such as cereals (European Food Safety Authority 2004), this study has the particularity that the influence of ZEN in a commonly used feedstuff can be investigated with only low background exposure to DON.

The aim of the present study was therefore to investigate the effect of molassed sugar beet pulp contaminated with elevated levels of ZEN and low levels of DON under standardized conditions in a commercial reproductive sow unit. The focus was on the zootechnical and reproductive performance of the sows. Additionally, the influence of ZEN exposure on the occurrence of lesions in sows and their piglets was investigated. All parameters were measured during the intake of ZEN-contaminated sugar beet pulp and additionally in the following lactation, without direct ZEN contamination via feed, in order to record any putative carry-over effects.

## Material and methods

The animal experiment was conducted according to the European Community regulations concerning the protection of experimental animals and the guidelines of the German Animal Welfare Act and was approved by the Lower Saxony State Office for Consumer Protection and Food Safety, Oldenburg, Germany (file number 33.19–42502-04–19/3253).

### Experimental design and diets

The study was designed as a dose–response trial including three different feed groups with increasing ZEN load in feed. The study lasted over two lactations (regardless of the parity of the sows), whereby an exposure phase in lactation 1 until day 40 after insemination was followed by a washout phase, which included the rest of gestation and lactation 2.

The study was carried out on a commercial farm with 1900 reproducing sows, which were divided into groups of 185 to 195 sows, so there was a total of ten production groups on the farm. The sows had Danish breeding genetics (Yorkshire x Landrace) and were inseminated with the semen of a Piétrain boar.

For the experiment, 90 randomly selected sows were included, taking care that sows were evenly distributed over parity 1 to 6. In addition, four male and four female piglets per sow and lactation were randomly selected on day 5 *post* *partum* and included in the study. Thirty sows were randomly assigned to one of three experimental groups considering parity. Each experimental group was provided with a diet containing different levels of ZEN (Table 1). The control group received a diet that was ZEN-free or as low in ZEN as possible (CON). Experimental group 1 received a diet with a target concentration of 250 µg ZEN/kg feed (88% DM; ZEN1), and experimental group 2 received a diet with a target concentration of 500 µg ZEN/kg feed (88% DM; ZEN2). The different concentrations of ZEN were obtained by including ZEN-contaminated or non-ZEN-contaminated molassed sugar beet pulp into the diets. The contaminated sugar beet pulp contained 2204.8 µg ZEN/kg DM, while the non-contaminated sugar beet pulp contained 107.3 µg ZEN/kg DM. In order to include the appropriate ZEN content in the experimental feed mixtures, 13% of the contaminated and/or non-contaminated sugar beet pulp was mixed into diets. The feed mixtures were similar to the mixtures commonly used on the commercial farm and were optimized accordingly on a cost basis. The study lasted over two lactations and started when sows were housed in the farrowing pen and thus with lactation 1. During lactation 1, sows were fed the usual lactation diet with the appropriate amount of ZEN-contaminated sugar beet pulp. Sows were housed in the farrowing pens, equipped with farrowing crates and feeding troughs, as well as nipple drinkers for sow and offspring, 3 days before the calculated farrowing date and stayed there for 25 days, after which they were transferred to the insemination area. There, sows were fed the usual gestation diet, with the appropriate ZEN concentration corresponding to their feeding group (CON, ZEN1, ZEN2). Forty days after insemination, an ultrasound was performed on sows for the final determination of pregnancy. From this point on, all sows were fed the same diet as pregnant sows, which did not contain ZEN-contaminated sugar beet pulp. Three days before the calculated farrowing date, sows were again housed in farrowing pens, where they were all fed the same lactation diet, which did not contain ZEN-contaminated sugar beet pulp (lactation 2). The study ended when sows were moved out of the farrowing pen after lactation 2.

**Table 1:** Ingredients (g/kg as fed) of diets for sows fed with control diet (CON) and diets with targeted zearalenone (ZEN) concentrations of 250 µg/kg (ZEN1) and 500 µg/kg (ZEN2)

	Gestation feed (d 74 to 3 a.p.)	Lactation feed (lactation 1; d 1 to 25 p.p.)			Gestation feed (d 26 to 66 p.p.)			Lactation feed (lactation 2; d 1 to 25 p.p.)
		CON	ZEN1	ZEN2	CON	ZEN1	ZEN2	
Barley	490	260	260	260	500	500	500	285
Wheat	120	280	280	280	100	100	100	280
Molasses sugar beet pulp (low ZEN concentration)	75	130	65	–-	130	65	–-	35
Molasses sugar beet pulp (high ZEN concentration)	–-	–-	65	130	–-	65	130	–-
Wheat bran	130	22	22	22	80	80	80	120
Soybean extraction meal	19	50	50	50	30	30	30	40
Rapeseed extraction meal	18	25	25	25	20	20	20	25
Soybeans (steam heated)	19	90	90	90	19	19	19	70
Corn		45	45	45				45
Whey concentrate	20	9	9	9	18	18	18	9
Linseed extruded	17	19	19	19	17	17	17	19
Palm kernel extraction meal	13				12	12	12	
Brewer’s yeast	11	10	10	10	11	11	11	10
Soy hulls	17				11	11	11	
Sunflower extraction meal	17	13	13	13	11	11	11	18
Calcium carbonate	10	7	7	7	8	8	8	13
Refining fatty acids	4	8	8	8	5	5	5	7
Monocalcium phosphate	2	8	8	8	3	3	3	5
Sodium bicarbonate	4				3	3	3	
Sodium chloride	2	5	5	5	3	3	3	6
Magnesium oxide	1				1	1	1	
Premix^1,2^	11	19	19	19	18	18	18	13

^1^Nutritional supplements per kg lactation feed: 12,000 I.U. vitamin A, 2000 I.U. vitamin D3, 135 mg vitamin E/all rar-alpha tocopheryl acetate, 500 µg biotin, 200 mg iron, 60 mg manganese, 90 mg zinc, 4.4 mg iodine, 0.4 mg selenium, 10 mg copper, 0.03% hydroxy analogon of methionine

^2^Nutritional supplements per kg gestation feed: 12,000 I.U. vitamin A, 2000 I.U. vitamin D3, 140 mg vitamin E/all rar-alpha tocopheryl acetate, 500 µg biotin, 200 mg iron, 60 mg manganese, 90 mg zinc, 4.4 mg iodine, 0.4 mg selenium, 10 mg copper, 0.03% hydroxy analogon of methionine

### *Sample collection (*Table 2*)*

**Table 2:** Overview of the experiment, sampling, and procedures with differentiation of control diet (CON), diet with a targeted concentration of zearalenone of 250 µg/kg (ZEN1), and diet with targeted concentrations of ZEN of 500 µg/kg (ZEN2) for lactation feed during lactation 1 and gestation feed and presentation of collected data and samples

Phase	Day	Time during reproduction schedule	Feed	Number of sows	Number of piglets	Samples
Gestation	− 3	Stabling in the farrowing pen	Gestation	90	-	Back fat thickness, body condition score, weight, blood (biochemistry)
Lactation 1	1	Day 1 p.p	CON	30	240	Number of total/alive/dead born piglets
			ZEN1	30	240	
			ZEN2	29	232	
	5	Day 5 p.p	CON	30	240	Litter weight; lesions
			ZEN1	30	240	
			ZEN2	29	232	
	18	Day 18 p.p	CON	30	240	Back fat thickness, body condition score weight, blood (biochemistry); litter weight; lesions
			ZEN1	30	240	
			ZEN2	29	232	
	24	Weaning	CON	30	240	Number of weaned piglets
			ZEN1	30	240	
			ZEN2	29	232	
Gestation	64	2 ultrasounds after insemination	CON	30		Blood (biochemistry, ZEN exposure)
			ZEN1	30		
			ZEN2	29		
	138	Stalling in the farrowing pen	Gestation	83		Back fat thickness, body condition score weight
Lactation 2	142	Day 1 p.p	Lactation	83	664	Number of total/alive dead born piglets
	147	Day 5 p.p	Lactation	83	664	Litter weight; lesions
	157	Day 18 p.p	Lactation	83	664	Back fat thickness, body condition score weight; blood (biochemistry); litter weight; lesions
	165	Weaning	Lactation	83	664	Number of weaned piglets

#### Feed samples

Both the lactation diet and gestation diet, into which contaminated and non-contaminated sugar beet pulp had been mixed, were sampled daily. A pooled sample was formed and analyzed according to the methods of the Association of German Agricultural Analytic and Research Institutes (VDLUFA 1988). Dry matter, ether extract, crude fiber, and crude ash were analyzed according to methods 3.1, 5.1.1, 6.1.1, and 8.1, respectively. Furthermore, acid detergent fiber and neutral detergent fiber were determined using methods 6.5.2 and 6.5.1, respectively. Starch in the diet was analyzed polarimetrically according to method number 7.2.1, and sugar was determined according to method 7.1.1. Additionally, both batches of sugar beet pulp and all diets were analyzed with regard to their concentration of ZEN, as well as DON. Furthermore, the supplementary diets given to the piglets during the suckling period from day 3 after birth (piglet milk, pre-starter 1, pre-starter 2) were examined with regard to nutrient and toxin concentration.

The feed intake of the sows was recorded on a weekly basis at the group level during lactation 1 and the gestation phase since feed was manually presented during these periods. During the high gestation phase and lactation 2, the amount of ingested feed by sows as well as the amount of ingested feed by piglets could not be recorded since feeding was automatic.

#### Performance and reproduction

Sows were weighed at the time of stalling in and out of the farrowing pen. At these times, body condition was examined using a five-level score according to Schrader et al. (2016), and backfat thickness was measured at the last rib 0.6 cm beside the backbone using an ultrasound indicator (Renco lean meater® indicator). Immediately after birth, the number of dead, live, and total born piglets per sow was recorded. Over 3 days, the litters within the experimental groups were balanced with respect to the number of piglets per litter, as is usual on the farm. On day 5 after birth and thus after balancing the litters, the weight of the litter per sow was recorded. On day 25 *post* *partum* at weaning, the number of weaned piglets per litter and the litter weight were recorded.

#### Blood samples

Blood samples were drawn by venous puncture (*V. cava cranialis sive brachiocephalica*) according to standard veterinary practice and collected into serum tubes and left clotting for 60 min at room temperature. Samples were centrifuged at 1952 × g for 15 min (15 °C), and serum was frozen at − 18 °C*.* Up to this point, the samples were prepared directly on the farm. In the laboratory, samples were stored at − 80 °C until further processing. In lactation 1, blood samples were taken from sows at stabling into the farrowing pen, as well as on day 18 ± 1 day (consequently day 18) *post* *partum* (p.p.) after farrowing. In addition, a blood sample was taken on day 40 after insemination. In lactation 2, blood samples were taken on day 18 p.p*.* Blood samples were analyzed for clinical-chemical parameters (total protein, albumin, urea, creatinine, aspartate-aminotransferase, alanine-aminotransferase, gamma-glutamyltransferase, total and direct bilirubin, alkaline phosphatase, glucose, triglycerides, cholesterol, chloride, sodium, potassium, calcium, phosphorus), which were measured on an automated analyzer (Indiko plus, Thermo Scientific, Henningsdorf, Germany) using the manufacturers reagent packs (Thermo Fisher Scientific, Vantaa, Finland). Non-esterified fatty acids (FUJIFILM Wako Chemicals Europe GmbH, Neuss, Germany), beta-hydroxybutyrate (DiaSys Diagnostic Systems GmbH, Holzheim, Germany), and glutamate dehydrogenase (Labor + Technik Eberhard Lehmann GmbH, Berlin, Germany) were analyzed on the same analyzer using reagent packs of the indicated manufacturers. In addition, the concentration of ZEN was determined using high-performance liquid chromatography-mass spectrometry/mass spectrometry (HPLC–MS/MS) multi-mycotoxin-method according to Brezina et al. (2014) in the blood sample 40 days after insemination to determine ZEN exposure.

#### Lesions

Sows and their offspring—four males and four females per sow and litter (if present)—were scored for lesions on day 5 and day 18 p.p*.* In sows, lesions in the form of scratches, redness, swellings on the mammary complex, overlong claws, and sole injuries were scored according to Schrader et al. (2016). Different gradations were recorded depending on the severity (score 0/1/2). The right and left sides were recorded separately. The absence or presence (score 0/1) of the following lesions according to Friedrich-Loeffler-Institut (2016) was recorded for each piglet: injury, necrosis, loss of length or partial loss of tail or ears, and swelling and/or redness on the vulva or mammary complex. As the scoring was done by several persons, training was carried out in advance using pictures and animals on the farm. The prevalence-adjusted bias-adjusted kappa (PABAK) was calculated to evaluate the agreement (Fleiss et al. 2003). If a PABAK of at least 0.6 was achieved, the agreement was accepted as sufficient (Fleiss et al. 2003). The scoring was carried out unblinded with regard to the feeding group.

### Statistical analyses

The analysis of changes in live weight and backfat thickness of the sows was performed separately for both lactations and parametrically using a mixed linear model and a Tukey–Kramer post hoc procedure, with the experimental group, the time point and the interaction of the experimental group, and time point as fixed factors and parity as a random factor in the model (proc mixed). The covariance structure was determined considering the Akaike Information Criterion (AIC). The same procedure was used for the analysis of data on the number of piglets born alive or dead and the total, the total number of weaned piglets, and the weight of litters, whereby the lactation, the experimental group, and the interaction of both were used as fixed factors. Likewise, the clinical chemistry data were evaluated using a linear model, with the analyzed concentration at day − 3 as a covariate in each case, the time point and the interaction of the experimental group and time point as fixed factors, and parity as a random factor in the model. A relation between the experimental group and the body condition score was tested using Fisher’s exact test. For the evaluation of the sows’ scores, the gradings were summarized in two groups (0/1; not present/present). In addition, the differentiation of the sides was postponed, and one value was formed for each sow (worst score as the basis). The prevalence of the presence of a lesion was calculated for both the sows and the piglets by dividing the number of affected animals by the number of all animals in a group. This resulted in one value per group for the sows so that these could only be considered descriptively. For the piglets, one value per sow was calculated so that a linear model with group and day and their interaction as fixed effects could then be calculated.

All analyses were performed in SAS 9.4, and results with a *p*-value ≤ 0.05 were assumed to be significant.

## Results

At the start of the trial, there were 30 sows in each group. During the trial, sows dropped out at various times. In group CON, two sows failed to become pregnant, this was determined at day 40 after insemination. In group ZEN1, one sow had to be put down at the end of lactation 1 due to a severe injury of the back. In group ZEN2, four sows were removed from the study. One sow (gilt) refused to feed. Since the sow was already conspicuously thin at the beginning of the trial, the sow’s data were not included in the evaluation. Additionally, one sow did not become pregnant and was removed from the trial on day 40 after insemination. One sow had to be put down during the gestation phase on day 26 due to a severe injury of the legs, and another sow died during the gestation phase on day 151 after the start of the trial. No sow had to be removed from the trial as a result of the experimental treatments (Table 3).

**Table 3:** Number of sows (losses) in three experimental groups differing in zearalenone concentration (targeted concentration: CON = 0 ZEN/kg feed, ZEN1 = 250 ZEN/kg feed, ZEN 2 = 500 µg ZEN/kg feed) differentiated by parity of sows

Parity	CON	ZEN1	ZEN2
1 (gilts)	5	5	4 (1)
2	5	6	5
3	5 (1)	5	5
4	5	5 (1)	5
5	5	4	5 (1)
6	3	3	3
7	2 (1)	2	2 (1)
Total	30 (2)	30 (1)	29 (3)

### Diet

In both experimental diets containing contaminated sugar beet pulp (lactation 1 and gestation until day 40 after insemination), the targeted gradations between the three experimental groups could be reproduced. Both the diet during gestation after day 40 after insemination and the diet in lactation 2 were only contaminated with low levels of ZEN (Table 4). The DON concentration was at a similarly low level in all rations for the sows.

**Table 4:** Analyzed nutrient concentration (g/kg, 88% DM) of diets for sows fed during study-oriented on-farm feeding regimen with differentiation of control diet (CON), diet with a targeted concentration of zearalenone of 250 µg/kg (ZEN1), and diet with a targeted concentration of ZEN of 500 µg/kg (ZEN2) for lactation feed during lactation 1 and gestation feed and diets for suckling piglets (piglet milk, pre-starter 1, pre-starter 2)

	Gestation feed (d 74 to 3 a.p*.*)	Lactation feed (lactation 1; d 1 to 25 p.p.)			Gestation feed (d 26 to 66 p.p.)			Lactation feed (lactation 2; d 1 to 25 p.p.)	Piglet milk	Prestarter 1	Prestarter 2
		CON	ZEN1	ZEN2	CON	ZEN1	ZEN2				
Dry matter (%)	89.46	88.06	88.08	88.20	87.72	87.66	87.68	88.77	19.95	92.97	91.20
Crude protein	177.06	180.69	180.28	182.24	155.78	160.06	155.88	181.14	925.61	204.34	194.46
Crude ash	60.62	63.81	65.00	60.12	60.33	59.75	57.17	63.29	250.06	55.56	51.73
Ether extract	57.51	58.48	59.00	60.40	46.83	49.13	49.00	61.17	390.99	116.64	84.13
Crude fiber	55.80	65.86	66.40	66.79	79.29	79.29	85.90	63.91	107.67	13.36	22.06
Neutral detergent fiber	193.63	200.11	187.64	190.74	237.83	258.17	251.69	214.16	-	86.03	103.34
Acid detergent fiber	74.50	83.74	82.61	86.52	99.24	106.42	110.10	79.43	-	18.87	33.68
Starch	411.15	421.80	421.79	414.33	416.01	413.03	414.60	415.81	795.57	237.16	404.27
Sugar	47.09	51.47	54.55	55.00	48.84	47.73	48.21	43.42	-	192.93	98.11
ME (MJ/kg)^1^	12.94	12.59	12.54	12.64	11.78	11.85	11.62	12.71	-	-	-
ZEN (µg/kg)	8.5	20.9	151.3	313.0	36.8	259.8	428.3	9.1	0.00	4.1	1.6
DON (µg/kg)	39.6	51.4	55.0	89.4	66.0	71.5	71.7	131.4	6.3	142.1	34.1

^1^Metabolisable energy (MJ/kg) = 0.021503 * crude protein (g/kg) + 0.032497 * ether extract (g/kg) − 0.021071 * crude fiber (g/kg) + 0.016309 * starch (g/kg) + 0.014701 * organic residue (g/kg) where organic residue = dry matter (g/kg) − (crude ash (g/kg) + crude protein (g/kg) + ether extract (g/kg) + crude fiber (g/kg) + starch (g/kg)), according toBulang and Rodehutscord (2009)

### Reproductive and zootechnical performance

**Fig. 1:**
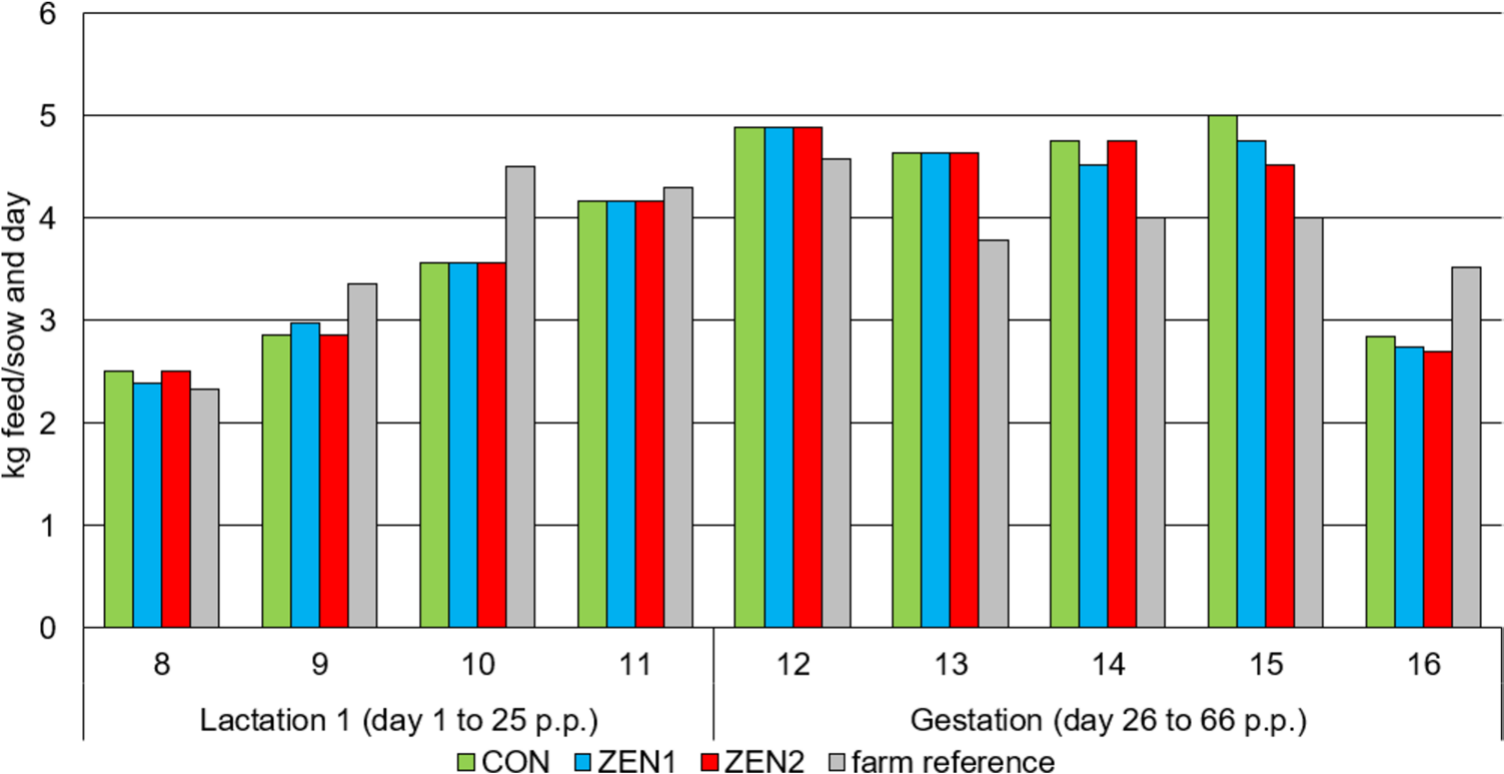
Mean daily feed intake (kg) per calendar week of sows’ fed diets with increasing ZEN concentrations (targeted concentration: CON = 0 µg ZEN/kg feed, ZEN1 = 250 µg ZEN/kg feed, ZEN 2 = 500 µg ZEN/kg feed) and farm reference per week during lactation 1 and gestation until day 40 after insemination (ZEN exposure phase)

When feeding the experimental diets (lactation 1 and gestation), the amount of feed per group was recorded so that the average feed quantity per sow and day could be calculated (Fig. 1). During lactation 1, the sows consumed between 2.3 and 4.2 kg feed/sow and day, which largely corresponded to the feed quantity of the rest of the sow herd. During the gestation phase, feed consumption was between 2.7 and 5.0 kg/sow and day, which also corresponds to the feed quantity of the rest of the sow herd. A group effect could not be seen. However, the feed quantities increased significantly during lactation and then decreased markedly during the gestation period.

**Fig. 2:**
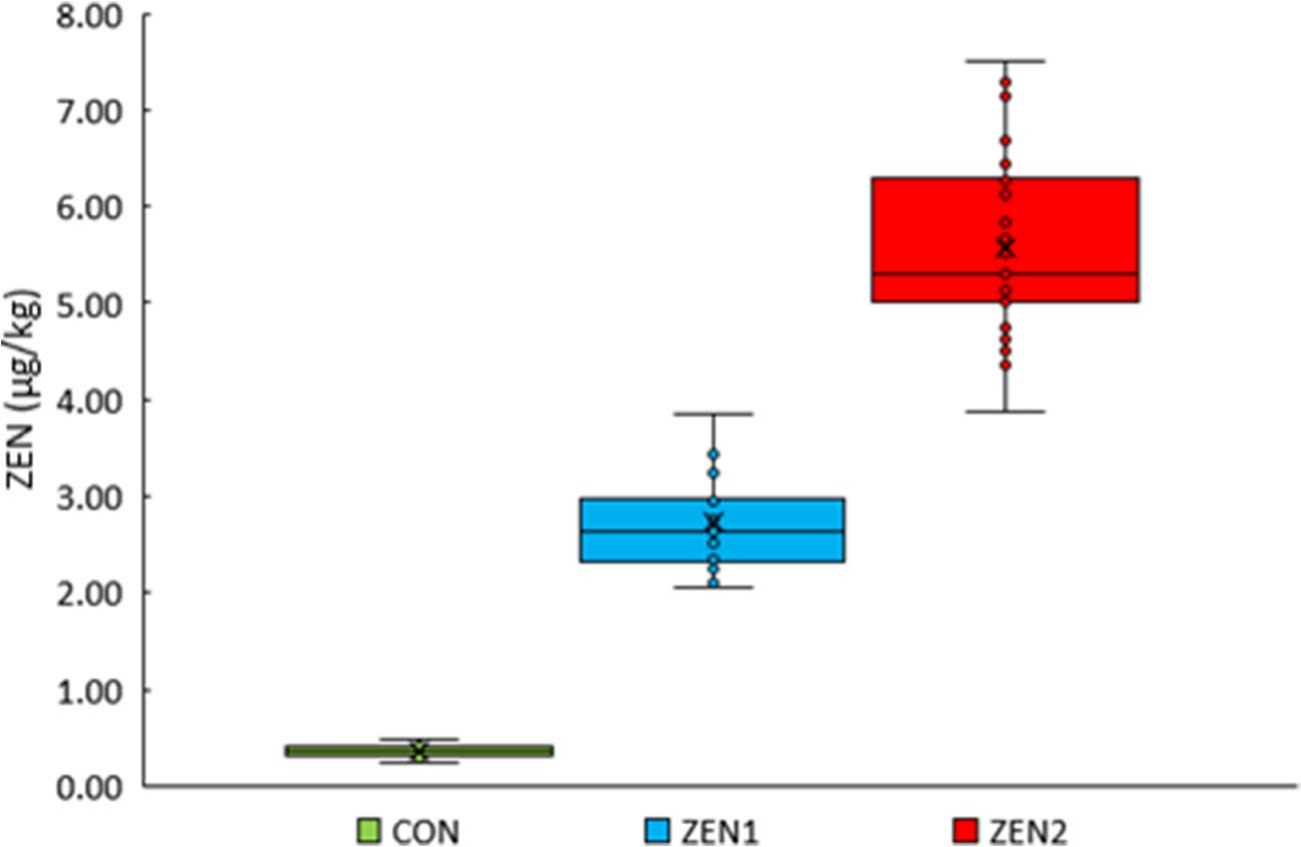
Dietary zearalenone (ZEN) exposure (µg ZEN/kg live weight) of sows’ fed diets with increasing ZEN concentrations (targeted concentration: CON = 0 µg ZEN/kg feed, ZEN1 = 250 µg ZEN/kg feed, ZEN 2 = 500 µg ZEN/kg feed) at day 18 ± 1 postpartum in lactation 1 and gestation until day 40 after insemination (ZEN exposure phase)

The dietary ZEN intake per kg live weight at day 18 p.p*.* differed significantly between groups (*p* < 0.001). In ZEN2, the dietary ZEN intake was the highest (mean ± SD: 5.57 ± 0.92 µg ZEN/kg live weight); in CON, the lowest (0.36 ± 0.06 µg ZEN/kg live weight); and in ZEN1, medium values were reached (2.73 ± 0.47 µg ZEN/kg live weight; Fig. 2).

**Fig. 3:**
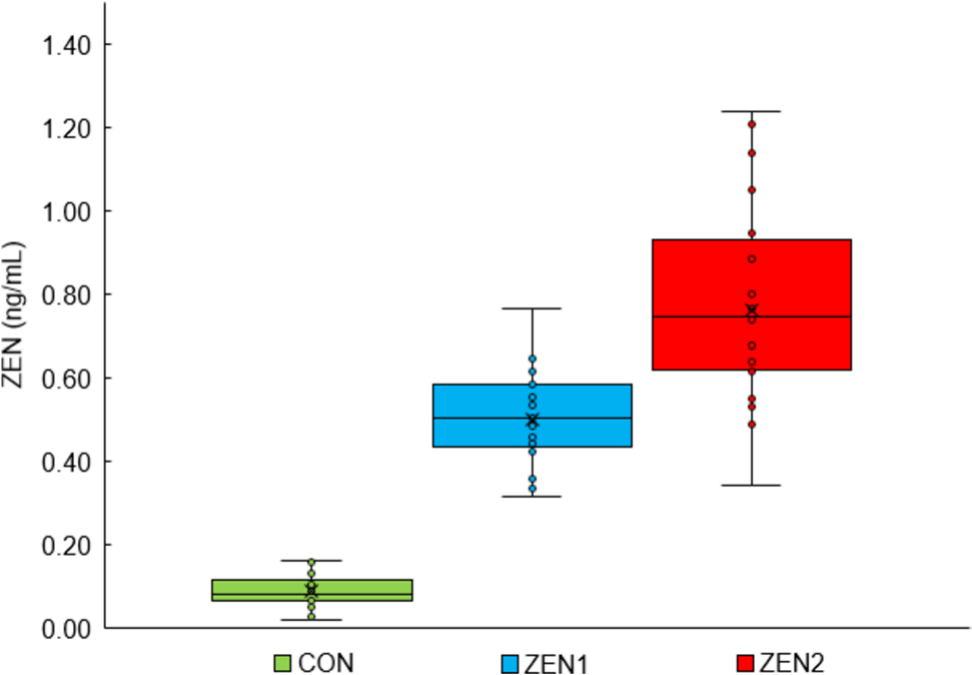
Zearalenone (ZEN) concentration in sows’ blood-fed diets with increasing ZEN concentrations (targeted concentration: CON = 0 µg ZEN/kg feed, ZEN1 = 250 µg ZEN/kg feed, ZEN 2 = 500 µg ZEN/kg feed) at the end of zearalenone exposure (day 40 after insemination)

The dietary ZEN exposure was clearly reflected in its ZEN plasma levels as a biological correlate (ZEN residue levels in blood at day 40 after insemination, Fig. 3). The serum concentration of ZEN differed significantly between all three groups (*p* < 0.001), where ZEN2 had the highest concentration (mean ± SD, 0.76 ± 0.22 µg/L) and CON (0.09 ± 0.04 µg/L) the lowest, while ZEN1 had a mean concentration (0.50 ± 0.11 µg/L).

In lactation 1, the live weight and backfat thickness of sows did not differ between the groups on either day − 3 before farrowing or day 18 after farrowing. In contrast, there was a reduction in both parameters from day − 3 to 18 p.p*.* irrespective of treatment. In lactation 2, the live weight of sows decreased again significantly from day − 3 to 18 p.p*.*, a group effect could not be found. The backfat thickness did not vary between time points or between groups (Table 5).

**Table 5:** Live weight and back fat thickness of sows (LS-Means ± SE) at day − 3 before farrowing (d-3) and day 18 ± 1 day (d18) p.p*.* fed lactation diet with different levels of zearalenone (lactation 1; targeted concentration: CON = 0 µg ZEN/kg feed, ZEN1 = 250 µg ZEN/kg feed, ZEN 2 = 500 µg ZEN/kg feed) or lactation diet with same level of zearalenone (lactation 2)

	d-3			d18			*p*-values	
	CON	ZEN1	ZEN2	CON	ZEN1	ZEN2		
Lactation 1								
Live weight (kg)	296.3 ± 7.3	286.6 ± 7.3	294.0 ± 7.4	246.2 ± 7.3	237.8 ± 7.3	243.0 ± 7.4	Day	** < 0.0001**
							Group	0.4442
							Day *group	0.9884
Back fat thickness (mm)	18.0 ± 0.8	16.4 ± 0.8	17.5 ± 0.8	15.4 ± 0.8	14.1 ± 0.8	14.9 ± 0.8	Day	**0.0001**
							Group	0.1874
							Day*group	0.9763
Lactation 2								
Live weight (kg)	313.8 ± 6.8	316.7 ± 6.7	315.5 ± 7.0	279.2 ± 7.0	275.9 ± 6.7	274.6 ± 7.1	Day	** < 0.0001**
							Group	0.9734
							Day*group	0.8716
Back fat thickness (mm)	18.7 ± 0.9	19.4 ± 0.9	18.0 ± 0.9	17.9 ± 0.9	17.2 ± 0.9	16.9 ± 0.9	Day	0.0624
							Group	0.5310
							Day*group	0.7166

**Fig. 4:**
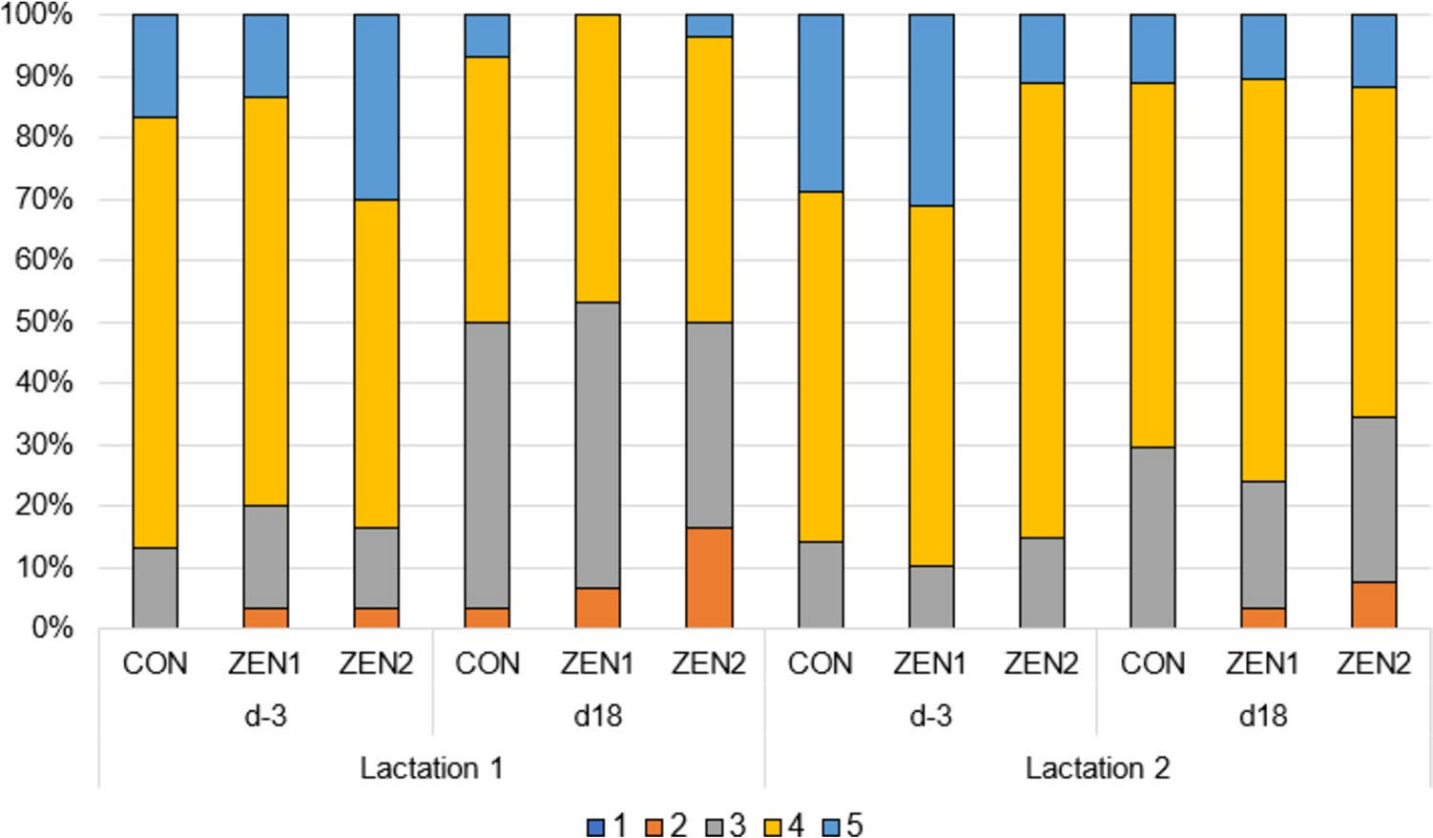
Distribution of body condition score in sows (1 = emaciated; 5 = overly fat) at day − 3 before farrowing (d-3) and day 18 ± 1 day (d18) postpartum fed lactation diet with different levels of zearalenone (lactation 1; targeted concentration: CON = 0 µg ZEN/kg feed, ZEN1 = 250 µg ZEN/kg feed, ZEN 2 = 500 µg ZEN/kg feed) or lactation diet with same level of zearalenone (lactation 2)

In both lactations 1 and 2, between 67 and 90% of sows had a body condition score of 3 or 4 and were thus within the normal range (Fig. 4). It can be seen that the proportion of fat (score 4) and overly fat (score 5) sows decreased in both lactations from day − 3 to day 18 p.p*.*, while the number of ideal (score 3) and thin (score 2) sows increased. A significant relation between the experimental group and the body condition score could not be established at any time (*p*-value: lactation 1, day − 3, 0.654/day 18, 0.458; lactation 2, day − 3, 0.418/day 18, 0.880).

There were no abnormalities in the sows during insemination. The number of inseminations did not deviate from the farm average (1 to 3 inseminations per sow). In CON and ZEN2, two and one sows, respectively, did not become pregnant, which is for CON quite higher than the average for the farm (mean proportion of sows returning to service on the farm, 2.3%).

Neither the number of total born piglets nor the number of live, dead, or weaned piglets differed between groups or lactations. Litter weight was on both days in lactation 2 significantly lower than in lactation 1, a significant difference between groups could not be found in both lactations. Consequently, the litter weight at day 18 p.p*.* was in lactation 2 significantly lower than in lactation 1, also without a difference between the groups. There was a significant interaction between lactation and group for litter weight gain. Litter weight gain of ZEN2 in lactation 1 was significantly higher than all other groups in lactation 1 and 2 (Table 6).

**Table 6:** Number of total, live and stillborn born piglets, and weaned piglets (LS-Means ± SE) as well as litter weight at day 5 and day 18 p.p*.* and litter weight gain for sows fed diets with different levels of zearalenone (lactation 1; targeted concentration: CON = 0 ZEN/kg feed, ZEN1 = 250 ZEN/kg feed, ZEN 2 = 500 µg ZEN/kg feed) or lactation diet with same level of zearalenone (lactation 2)

	Lactation 1			Lactation 2			*p*-value	
	CON	ZEN1	ZEN2	CON	ZEN1	ZEN2		
Total born piglets	20.5 ± 0.8	20.6 ± 0.8	19.2 ± 0.8	21.5 ± 0.8	20.4 ± 0.8	19.9 ± 0.9	Lactation	0.4867
							Group	0.2109
							Lactation*group	0.7795
Live born piglets	17.0 ± 0.7	16.3 ± 0.7	16.7 ± 0.7	18.6 ± 0.7	17.0 ± 0.7	16.7 ± 0.7	Lactation	0.1759
							Group	0.1695
							Lactation*group	0.4880
Stillborn piglets	3.3 ± 0.5	3.8 ± 0.5	2.5 ± 0.5	2.6 ± 0.4	2.9 ± 0.5	3.0 ± 0.5	Lactation	0.0691
							Group	0.6053
							Lactation*group	0.2138
Weaned piglets	13.4 ± 0.2	13.2 ± 0.2	13.6 ± 0.2	12.9 ± 0.2	13.1 ± 0.2	13.1 ± 0.3	Lactation	0.0630
							Group	0.5409
							Lactation*group	0.5386
Litter weight d5 (kg)	25.7 ± 0.7	25.4 ± 0.7	25.0 ± 0.7	23.0 ± 0.7	23.3 ± 0.7	21.2 ± 0.8	Lactation	**0.0002**
							Group	0.8913
							Lactation*group	0.8247
Litter weight d18 (kg)	63.2 ± 1.8	61.9 ± 1.8	68.2 ± 1.8	59.3 ± 1.9	61.3 ± 1.9	59.4 ± 2.0	Lactation	**0.0042**
							Group	0.3312
							Lactation*group	0.0886
Litter weight gain (kg)	37.5 ± 1.5^a^	36.5 ± 1.5^a^	43.2 ± 1.5^b^	36.3 ± 1.6^a^	38.0 ± 1.5^a^	35.0 ± 1.6^a^	Lactation	0.0348
							Group	0.3097
							Lactation*group	**0.0049**

Different superscripts indicate significant differences

### Clinical biochemistry

A significant time effect was found for almost all clinical-chemical parameters in sows (Supplemental Tables S1–4): differences occurred not only between the different phases of gestation (day − 3 p.p*.* high gestation, day 18 p.p*.* lactating, day 40 after insemination gestation) but also between day 18 p.p*.* in lactations 1 and 2. Total protein showed no impact on group or time. Only three parameters showed a significant effect of group or a significant interaction between group and time: albumin, urea, and glucose. Albumin concentration not only varied significantly between the time points but also between groups, with ZEN1 (LS-means ± SE, 37.72 ± 0.22 g/L) having a significantly lower concentration than ZEN2 (38.45 ± 0.23 g/L; *p* = 0.0538), while CON did not differ from both ZEN-groups (37.74 ± 0.22 g/L). A significant interaction between group and time was found for the urea concentration in lactation 2, and urea concentration in ZEN2 (3.30 ± 0.14 mmol/L) was significantly lower than in ZEN1 (4.04 ± 0.14 mmol/L; *p* = 0.0132) on day 18 p.p*.*, but CON did differ from the other groups (3.76 ± 0.14 mmol/L). In addition, glucose concentration also had a significant interaction between group and time: on day − 3 p.p*.* in lactation 1, glucose was significantly higher in ZEN2 (4.21 + 0.09 mmol/L) than in CON (3.75 + 0.09 mmol/L; *p* = 0.0155), whereas ZEN1 glucose concentration was between the other groups (3.83 ± 0.09 mmol/L). Beta-hydroxybutyrate was also analyzed in blood samples, but all concentrations were below the limit of detection (0.096 mmol/L), so no further analysis was carried out with these data.

### Lesions of sows and their offspring

The scoring of the sows’ mammary complexes did not produce a clear result (Table 7). While there was an increase in the prevalence from day 5 to day 18 p.p*.* in lactation 1, the prevalence decreased in lactation 2. A clear difference between the groups could not be determined either. The prevalence of overlong claws in both lactations was higher on day 5 than on day 18 p.p*.* in all groups, whereby the claws were shortened by the farmer during the suckling phase. The prevalence of sole injuries was relatively high in lactation 1 on both day 5 and day 18 p.p. In lactation 2, sole injuries were less prevalent in all groups on day 5 p.p*.*, with a sharp increase on day 18 p.p*.*

**Table 7:** Lesion prevalence (%) of sows fed diets with different levels of zearalenone (lactation 1; targeted concentration: CON = 0 ZEN/kg feed, ZEN1 = 250 ZEN/kg feed, ZEN 2 = 500 µg ZEN/kg feed) or lactation diet with the same level of zearalenone (lactation 2) on day 5 (d5) and day 18 (d18) postpartum

		Mammary complexes			Overlong claws			Sole injuries		
		CON	ZEN1	ZEN2	CON	ZEN1	ZEN2	CON	ZEN1	ZEN2
Lactation 1	d5	25.0	50.0	27.6	64.0	56.0	58.6	71.4	67.9	76.7
	d18	50.0	46.7	56.7	33.3	33.3	33.3	86.7	93.3	86.7
Lactation 2	d5	63.0	75.9	70.4	96.4	79.3	88.9	17.9	34.5	33.3
	d18	42.9	48.3	50.0	28.6	31.0	38.5	96.4	93.1	84.6

In lactation 1, there was a significant increase in the prevalence of tail and ear injuries and ear necrosis in piglets from day 5 to day 18 p.p*.* In contrast, lesions on the vulva and mammary complexes were significantly less prevalent on day 18 p.p*.* than on day 5 p.p. A group effect was only found in the prevalence of ear injuries, with the prevalence in ZEN2 being significantly higher than in ZEN1 and CON (Table 8). In lactation 2, there was a group-day interaction effect on tail and ear injuries, with the prevalence of these injuries increasing significantly from day 5 to day 18 p.p*.* While there was no significant difference between groups on day 5 p.p*.*, the prevalence of tail injuries on day 18 p.p*.* was significantly higher in ZEN2 than in ZEN1 and CON. Similarly, there was no significant difference in the prevalence of ear injuries between groups on day 5 p.p. In contrast, ear injuries on day 18 p.p*.* were significantly more prevalent in CON than in ZEN1 and ZEN2. The prevalence of lesions on mammary complexes was significantly lower on day 18 than on day 5 p.p*.* (Table 8).

**Table 8:** Lesion prevalences (%) of four male and four female piglets per litter (LS-Means ± SE) of sows fed diets with different levels of zearalenone (ZEN) (lactation 1; targeted concentration: CON = 0 ZEN/kg feed, ZEN1 = 250 ZEN/kg feed, ZEN 2 = 500 µg ZEN/kg feed) or lactation diet with same level of zearalenone (lactation 2) recorded at day 5 p.p*.* (d5) and day 18 ± 1 p.p*.* (d18)

		d5			d18			*p*-value	
		CON	ZEN1	ZEN2	CON	ZEN1	ZEN2		
Lactation 1									
Tail	Injury	4.0 ± 2.4	6.6 ± 2.4	5.5 ± 2.4	10.7 ± 2.4	17.7 ± 2.4	18.9 ± 2.4	Day	** < 0.0001**
								Group	0.0702
								Day*group	0.3733
	Necrosis	0.0 ± 0.2	0.0 ± 0.2	0.0 ± 0.2	0.0 ± 0.2	0.0 ± 0.2	0.5 ± 0.2	Day	0.3187
								Group	0.3699
								Day*group	0.3700
	Loss	-	-	-	-	-	-	Day	
								Group	
								Day*group	
Ears	Injury	42.6 ± 5.4	56.4 ± 5.4	54.2 ± 5.2	62.0 ± 5.4	68.8 ± 5.2	87.2 ± 5.4	Day	** < 0.0001**
								Group	**0.0039**
								Day*group	0.1633
	Necrosis	0.0 ± 0.7	0.0 ± 0.7	0.4 ± 0.7	1.3 ± 0.7	0.8 ± 0.7	1.7 ± 0.7	Day	**0.0420**
								Group	0.6478
								Day*group	0.9290
	Loss	-	-	-	-	-	-	Day	
								Group	
								Day*group	
Vulva	Swelling/redness	1.7 ± 0.8	2.9 ± 0.8	0.8 ± 0.8	0.0 ± 0.8	0.4 ± 0.8	0.8 ± 0.8	Day	**0.0259**
								Group	0.4884
								Day*group	0.2465
Mammary complexes	Swelling/redness	20.0 ± 2.8	12.9 ± 2.8	20.2 ± 2.8	6.0 ± 2.8	6.2 ± 2.8	2.5 ± 2.8	Day	** < 0.0001**
								Group	0.4261
								Day*group	0.1549
Lactation 2									
Tail	Injury	0.6 ± 1.8^a^	0.9 ± 1.8^a^	2.4 ± 1.8^ac^	15.8 ± 1.8^b^	14.7 ± 1.8^b^	5.6 ± 1.8^ac^	Day	< 0.0001
								Group	0.0460
								Day*group	**0.0023**
	Necrosis	-	-	-	-	-	-	Day	
								Group	
								Day*group	
	Loss	-	-	-	-	-	-	Day	
								Group	
								Day*group	
Ears	Injury	14.3 ± 3.9^a^	18.3 ± 3.9^a^	19.8 ± 4.0^a^	79.8 ± 3.9^b^	75.7 ± 3.9^bc^	61.5 ± 4.1^c^	Day	< 0.0001
								Group	0.1865
								Day*group	**0.0118**
	Necrosis	0.0 ± 0.1	0.1 ± 0.1	0.0 ± 0.1	0.0 ± 0.1	0.0 ± 0.1	0.1 ± 0.1	Day	0.2857
								Group	0.3289
								Day*group	0.3289
	Loss	-	-	-	-	-	-	Day	
								Group	
								Day*group	
Vulva	Swelling/redness	0.9 ± 0.5	0.4 ± 0.5	0.5 ± 0.5	0.4 ± 0.5	0.4 ± 0.5	0.5 ± 0.5	Day	0.7175
								Group	0.8682
								Day*group	0.8619
Mammary complexes	Swelling/redness	13.5 ± 2.6	18.5 ± 2.5	18.5 ± 2.6	1.8 ± 2.6	3.2 ± 2.5	1.5 ± 2.7	Day	** < 0.0001**
								Group	0.4378
								Day*group	0.5981

Different superscripts indicate significant differences

## Discussion

The present dose–response experiment aimed at testing whether the guidance value of 0.25 mg ZEN/kg is indeed still suitable for protecting sows from adverse ZEN effects. For this, a control diet with background ZEN contamination and a diet with a ZEN concentration twice as high as the guidance value was tested besides the dietary ZEN level representing the guidance value. The analysis of diets for ZEN revealed a good agreement between tested and targeted levels. However, ZEN concentration in gestation feed was slightly higher in all groups compared to lactation diets, although the same amount of sugar beet pulp was used for both. Thus, we can assume that additional ZEN contamination was realized by one of the other feed components. Nevertheless, the targeted gradation in ZEN contamination of diets was achieved. The gradual exposure of the sows to ZEN was well reflected in the blood of the sows. In contrast, the feeds during the last two-thirds of gestation and the feed in lactation 2 were only minimally contaminated with ZEN. The nutrient concentration between the groups was marginally different. The feeds in lactation 1 and 2 had partially different ingredients, but the nutrient concentration was only slightly different. The feed intake of the sows in all groups corresponded to the farm average in almost all weeks. An influence of the ZEN load, for example on palatability (Reddy et al. 2018), could not be determined. The supplementary feed for the piglets contained only small amounts of ZEN and was far below the guideline values (European Commission 2016). Therefore, the influence of ZEN ingested by the piglets via the supplementary feed can be neglected.

### Reproductive performance

Live weight and backfat thickness of sows in lactation 1 were lower than in lactation 2 due to the amount of gilts present in lactation 1. At the same time, it can be seen that the live weight of the sows decreased significantly in both lactations from day − 3 to day 18 p.p. This reduction can be explained on the one hand by the mass of the piglets and, on the other hand, by additional reproductive physiological tissues (e.g. placenta), which are shed after birth. Furthermore, milk production is associated with the mobilization of fat reserves, on the one hand, to provide nutrients for the milk, and on the other hand, the process of milk production is highly energy-intensive. This can also be illustrated by a reduced backfat thickness (Decaluwé et al. 2013), which decreased in both lactations. As neither live weight loss nor milk yield in this physiological state is influenced by ZEN, and the sows in the three test groups were fed feed with the same nutritional concentration, a group effect in these parameters was not to be expected.

No differences were found in the number of piglets born alive or dead between groups or between lactations. Contrary to comparable studies, there was no increase in the number of piglets born dead, nor was the total number of piglets born reduced as reported by Kanora and Maes (2009). Piglet mortality during the suckling phase does not tend to increase with ZEN-contaminated lactation feed (Benthem de Grave et al. 2021a), but this parameter could not be verified in our study because piglet mortality was not recorded due to litter balancing. Litter weight was significantly lower in lactation 2 than in lactation 1. This continued until weaning weight, which was also significantly lower in lactation 2 than in lactation 1. Litter weight is influenced by the body condition of sows (Ajay et al. 2023), genotype, or feeding (Zhukorskyi et al. 2023), among other things. As there was no difference between the groups, the reduced litter weight cannot be explained by the ZEN exposure. The increase in litter weight over the lactation did not differ significantly between the lactations, but the increase in litter weight in lactation 1 was significantly higher in the ZEN2 group than in all other groups in both lactations. Benthem de Grave et al. (2021b) have found that suckling piglets from sows fed with ZEN-contaminated feed had reduced glucagon-like peptide 1 (GLP1) levels in their serum. Reduced GLP1 concentration can result in higher feed intake due to the influence on the feeling of satiety, which in turn can lead to higher litter weight gain. In addition, it can be seen that the piglets in group ZEN2 have a slightly higher weight on day 18 p.p*.*, although this cannot be statistically verified.

### Clinical biochemistry

The significant increase in albumin, glucose, and calcium and the significant reduction in urea, creatinine, alanine aminotransferase, gamma-glutamyl transferase, triglycerides, cholesterol, potassium, and phosphorus from day − 3 to day 18 p.p*.* in the blood of the sows can be explained physiologically by the different reproductive states (day − 3 and day 40, gestating; day 18, lactating) and has also been described in other studies (Guo et al. 2019; Nitovski et al. 2011; Strathe et al. 2017; Verheyen et al. 2007). However, it is noticeable that a large part of the parameters also varied significantly on day 18 p.p*.* in both lactations, whereby sows are presumably in a comparable physiological state. Nevertheless, this variation might be explained by the proportion of gilts included in lactation 1 (Verheyen et al. 2007)—besides coping with their first pregnancy, gilts face the additional challenge of maturation and growth, which is completed during the second pregnancy in lactation 2. A significant difference in the glucose concentration between groups at day − 3 p.p*.* in lactation 1 is due to an unsatisfactory sampling, which was carried out according to groups (ZEN2, ZEN1, CON). The significant group effects found for albumin, urea, and glucose were small compared to the physiological range and were all within reference values (Verheyen et al. 2007). It can therefore be assumed that these group effects are physiologically irrelevant. On the contrary, the overall very low differences within the groups indicate a very high degree of homogeneity within the sows.

### Lesions

The effect of ZEN exposure on lesions in the sows’ mammary complexes could not be determined. The scoring showed that there was an increase in the prevalence of lesions on mammary complexes in the CON and ZEN2 groups in lactation 1. In contrast, the prevalence of ZEN1 was at the level of all groups on day 18 p.p*.* (around 50%) from day 5 p.p*.* onwards. The high prevalence already on day 5 p.p*.* could be an indication of more restless sows, lower milk yield, or swellings and redness caused by ZEN exposure. As the scoring data do not indicate which lesions occurred more frequently, the influencing factor cannot be conclusively clarified. In lactation 2, the prevalence decreased in all groups from day 5 to day 18 p.p*.* This reversed trend in prevalence is probably due to the proportion of primiparous sows in lactation 1 that have not yet had experience handling piglets and thus might have experienced slightly more difficulties in suckling their litter. As claws of sows were trimmed as a zootechnical measure by the farmer during lactation, it is not surprising that the prevalence of overlong claws decreased from day 5 to day 18 p.p. On the other hand, it was found that there was an increase in sole injuries in both lactations. This may be an indication that the floor in the crate was too rough (Norring et al. 2006). All in all, the ZEN exposure showed no clear directed effect on the prevalence of lesions in sows, but this may also be due to the many different influencing factors.

Neither tail nor ear losses were determined in the piglets. The prevalence of tail and ear injuries in piglets increased significantly in both lactations from day 5 to day 18 p.p. In the course of lactation, there is often increased biting of littermates when drinking at the teats or playing, which is often associated with bite injuries to tails and ears and thus an increase in prevalence (Gavaud et al. 2023). In addition, biting behavior can also occur as a reaction to an inflammatory process and the associated increase in cytokine production (Boyle et al. 2022; Nordgreen et al. 2020). In lactation 1, ear lesions were significantly more prevalent in ZEN2 than in CON. The same can be seen for the prevalence of tail injuries, although this can only be reported as a statistical trend. But at the same time, the sows had numerically more injuries on the mammary complexes. This could be an indication of increased hunger in the piglets, as ear and tail biting often occur when they are crowded around the teats. Additionally, piglets in ZEN2 showed an increased litter weight gain, which may be due to a reduced feeling of satiety caused by a reduced GLP1 level, as described above. At the same time, these effects were no longer seen in lactation 2, where the prevalence of ear injuries in ZEN2 increased the least toward day 18 p.p*.* and did not increase significantly at all in tail injuries. In lactation 1, the prevalence of ear necrosis increased significantly in all groups from day 5 to day 18 p.p*.* This indicates that the injuries caused by biting probably led to the development of necrosis (Reiner 2022). Since both injuries and necrosis of the tail and ear are multifactorial (Gavaud et al. 2023; Reiner 2022), it cannot be conclusively determined what led to the occurrence of the lesions.

In both lactations 1 and 2, redness and swelling of the mammary complexes of piglets were significantly less prevalent on day 18 than on day 5 p.p*.*; in lactation 1, this was also the case for redness and swelling of the vulva. This could be an indication of an inflammatory process possibly caused by a ZEN transfer from the sows to the piglets (Reiner 2022). However, the high prevalence occurred equally in all three groups, as well as in lactations 1 and 2, so an influence of ZEN can almost be ruled out. Since the prevalence was also significantly lower on day 18 p.p*.* in all three groups, the lesions were probably caused more by the stress of the birth and an associated inflammation or by the housing conditions (e.g., rough flooring).

## Conclusion

In commercial breeding sows exposed to dietary ZEN derived from sugar beet pulp, a clear dose–response pattern in serum at the end of exposure was demonstrated. There were very few discernible effects on the reproductive and zootechnical performance of sows or their offspring due to ZEN exposure: litter weight gain was significantly higher at the highest ZEN exposure, but this impact was not maintained during the subsequent lactation with control diets. Sow health traits such as clinical biochemistry also showed no clear effect of ZEN. The prevalence of ear and tail injuries in offspring of ZEN-exposed sows was increased during the exposure phase, but due to the multifactorial nature of such lesions in suckling piglets, it cannot be attributed unequivocally to ZEN exposure of their dams.

We did not see evidence in our data of a putative carry-over of ZEN from sow’s adipose tissue being released in a catabolic state, negatively affecting their piglets.

Overall, our results suggest that graded levels of dietary ZEN, even at double the guidance value (250, 500 µg/kg DM) appear to have only a minor impact on the reproductive performance of modern breeding sows and their offspring. Whether this is due to a lesser susceptibility to the estrogenic effects of ZEN in modern breeds or a different bioavailability and metabolization of ZEN originating from sugar beets in contrast to ZEN derived from cereal products remains a matter of future investigations.

## Supplementary information

Below is the link to the electronic supplementary material.

Supplementary file1 (DOCX 36 KB)

## Data Availability

.
