# Supplementary material for: Effect of zearalenone in sugar beet products on zootechnical and reproductive performance and lesions of sows and piglets
Source: Mycotoxin Res. 2024 Oct 12;41(1):47–61. doi: 10.1007/s12550-024-00564-z (PMC11757929; doi:10.1007/s12550-024-00564-z)
Supplement: Supplementary file 1 — Supplementary file1 (DOCX 36 KB) [file 12550_2024_564_MOESM1_ESM.docx]

Supplementary material

Table S1: Parameters of protein metabolism of sows (LSmeans ± SE) at day -3 (d-3) before farrowing, day 18 ± 1 day (d18) p.p*.* an day 40 (d40) after insemination fed the same gestation/lactation diet at d-3 and d18 in lactation 2 and fed lactation/gestation diet with different levels of Zearalenone (targeted concentration CON = 0 ZEN/kg feed, ZEN1 = 250 ZEN/kg feed, ZEN 2 = 500 µg ZEN/kg feed) in lactation 1 and gestation. Different superscripts indicate significant differences.

|  |  |  | Total protein [g/L] | Albumin [g/L] | Urea (mmol/L] | Creatinine [µmol/L] |
| --- | --- | --- | --- | --- | --- | --- |
| Lactation 1 | d-3 | CON | 76.52 ± 0.91 | 37.76 ± 0.43 | 5.70 ± 0.13^a^ | 233.07 ± 4.36 |
|  |  | ZEN1 | 75.72 ± 0.93 | 37.63 ± 0.44 | 5.48 ± 0.13^a^ | 237.84 ± 4.44 |
|  |  | ZEN2 | 75.46 ± 0.93 | 37.75 ± 0.44 | 5.37 ± 0.13^a^ | 234.28 ± 4.43 |
|  | d18 | CON | 73.49 ± 0.94 | 38.73 ± 0.44 | 3.10 ± 0.14^b^ | 203.31 ± 4.52 |
|  |  | ZEN1 | 75.49 ± 0.94 | 38.36 ± 0.44 | 3.61 ± 0.14^b^ | 209.16 ± 4.51 |
|  |  | ZEN2 | 75.86 ± 0.94 | 39.76 ± 0.44 | 3.55 ± 0.14^b^ | 217.69 ± 4.51 |
| Gestation | d40 | CON | 73.37 ± 0.94 | 37.70 ± 0.44 | 4.48 ± 0.14^c^ | 175.44 ± 4.51 |
|  |  | ZEN1 | 74.05 ± 0.94 | 38.00 ± 0.44 | 4.39 ± 0.14^c^ | 176.89 ± 4.51 |
|  |  | ZEN2 | 74.87 ± 0.96 | 38.86 ± 0.45 | 4.34 ± 0.14^c^ | 184.03 ± 4.59 |
| Lactation 2 | d18 | CON | 74.85 ± 0.95 | 36.76 ± 0.44 | 3.76 ± 0.14^de^ | 169.63 ± 4.51 |
|  |  | ZEN1 | 74.95 ± 0.94 | 36.88 ± 0.44 | 4.04 ± 0.14^d^ | 170.94 ± 4.52 |
|  |  | ZEN2 | 75.64 ± 1.00 | 37.44 ± 0.47 | 3.30 ± 0.14^e^ | 164.36 ± 4.78 |
| p-values | | | | | | |
|  |  | day | 0.1323 | **<0.0001** | <0.0001 | **<0.0001** |
|  |  | group | 0.4112 | **0.0313** | 0.0514 | 0.3152 |
|  |  | group*day | 0.6342 | 0.7607 | **0.0030** | 0.3726 |

Table S2a: Parameters of liver metabolism of sows (LSmeans ± SE) at day -3 (d-3) before farrowing, day 18 ± 1 day (d18) p.p*.* an day 40 (d40) after insemination fed the same gestation/lactation diet at d-3 and d18 in lactation 2 and fed lactation/gestation diet with different levels of Zearalenone (targeted concentration CON = 0 ZEN/kg feed, ZEN1 = 250 ZEN/kg feed, ZEN 2 = 500 µg ZEN/kg feed) in lactation 1 and gestation. Different superscripts indicate significant differences.

|  |  |  | Aspartate-aminotransferase [µkat/L] | Alanine-aminotransferase [µkat/L] | Aspartate-aminotransferase/ Alanine-aminotransferase ratio | Gamma-glutamyl transferase [µkat/L] | Total bilirubin [µmol/L] | Alkaline phosphatase [µkat/L] |
| --- | --- | --- | --- | --- | --- | --- | --- | --- |
| Lactation 1 | d-3 | CON | 1.09 ± 0.14 | 1.14 ± 0.03 | 0.88 ± 0.11 | 0.80 ± 0.02 | 3.15 ± 0.37 | 1.00 ± 0.05 |
|  |  | ZEN1 | 1.01 ± 0.14 | 1.11 ± 0.03 | 0.83 ± 0.12 | 0.78 ± 0.02 | 3.05 ± 0.38 | 0.96 ± 0.05 |
|  |  | ZEN2 | 1.39 ± 0.14 | 1.10 ± 0.03 | 1.18 ± 0.12 | 0.77 ± 0.02 | 3.18 ± 0.39 | 1.02 ± 0.06 |
|  | d18 | CON | 0.59 ± 0.15 | 0.70 ± 0.03 | 0.85 ± 0.12 | 0.56 ± 0.02 | 2.38 ± 0.39 | 0.88 ± 0.06 |
|  |  | ZEN1 | 0.68 ± 0.15 | 0.67 ± 0.03 | 0.85 ± 0.12 | 0.59 ± 0.02 | 2.61 ± 0.39 | 0.87 ± 0.06 |
|  |  | ZEN2 | 0.85 ± 0.15 | 0.70 ± 0.03 | 1.24 ± 0.12 | 0.57 ± 0.02 | 3.32 ± 0.39 | 0.91 ± 0.06 |
| Gestation | d40 | CON | 0.58 ± 0.15 | 1.06 ± 0.03 | 0.54 ± 0.12 | 0.76 ± 0.02 | 1.47 ± 0.41 | 0.88 ± 0.06 |
|  |  | ZEN1 | 0.59 ± 0.15 | 1.03 ± 0.03 | 0.51 ± 0.12 | 0.75 ± 0.02 | 1.28 ± 0.41 | 0.81 ± 0.06 |
|  |  | ZEN2 | 0.26 ± 0.15 | 0.97 ± 0.03 | 0.44 ± 0.12 | 0.73 ± 0.02 | 1.34 ± 0.41 | 0.64 ± 0.06 |
| Lactation 2 | d18 | CON | 0.70 ± 0.15 | 0.80 ± 0.03 | 0.75 ± 0.12 | 0.65 ± 0.02 | 1.97 ± 0.39 | 0.87 ± 0.06 |
|  |  | ZEN1 | 0.65 ± 0.15 | 0.78 ± 0.03 | 0.73 ± 0.12 | 0.63 ± 0.02 | 2.06 ± 0.39 | 0.91 ± 0.06 |
|  |  | ZEN2 | 0.55 ± 0.16 | 0.85 ± 0.03 | 0.63 ± 0.13 | 0.56 ± 0.03 | 2.52 ± 0.44 | 0.87 ± 0.06 |
| p-values | | | | | | | | |
|  |  | day | **<0.0001** | **<0.0001** | **<0.0001** | **<0.0001** | **<0.0001** | **<0.0001** |
|  |  | group | 0.9544 | 0.4491 | 0.2094 | 0.1550 | 0.3706 | 0.5024 |
|  |  | group*day | 0.1580 | 0.3701 | 0.1409 | 0.5435 | 0.8823 | 0.1631 |

Table S2b: Parameters of liver metabolism of sows (Median (Minimum | Maximum)) at day -3 (d-3) before farrowing, day 18 ± 1 day (d18) p.p*.* an day 40 (d40) after insemination fed the same gestation/lactation diet at d-3 and d18 in lactation 2 and fed lactation/gestation diet with different levels of Zearalenone (targeted concentration CON = 0 ZEN/kg feed, ZEN1 = 250 ZEN/kg feed, ZEN 2 = 500 µg ZEN/kg feed) in lactation 1 and gestation.

|  |  |  | Glutamate-dehydrogenase  [µkat/L] | Conjugated bilirubin [µmol/L] |
| --- | --- | --- | --- | --- |
| Lactation 1 | d-3 | CON | 0.03 (0.03 \| 0.06) | 0.99 (0.16 \| 5.39) |
|  |  | ZEN1 | 0.03 (0.03 \| 0.04) | 1.04 (0.60 \| 5.39) |
|  |  | ZEN2 | 0.04 (0.03 \| 0.33) | 1.00 (0.32 \| 17.40) |
|  | p-value | | 0.1304 | 0.4609 |
|  | d18 | CON | 0.03 (0.03 \| 0.22) | 1.02 (0.65 \| 1.98) |
|  |  | ZEN1 | 0.03 (0.03 \| 0.05) | 1.11 (0.70 \| 2.05) |
|  |  | ZEN2 | 0.04 (0.03 \| 0.25) | 1.06 (0.69 \| 5.41) |
|  | p-value | | 0.2537 | 0.5886 |
| Gestation | d40 | CON | 0.04 (0.03 \| 0.12) | 0.80 (0.59 \| 1.52) |
|  |  | ZEN1 | 0.05 (0.03 \| 0.10) | 0.80 (0.62 \| 1.14) |
|  |  | ZEN2 | 0.05 (0.4 \| 0.16) | 0.82 (0.56 \| 1.48) |
|  | p-value | | 0.1981 | 0.9520 |
| Lactation 2 | d18 | CON | 0.04 (0.03 \| 0.11) | 1.10 (0.86 \| 1.95) |
|  |  | ZEN1 | 0.04 (0.03 \| 0.25) | 1.17 (0.65 \| 1.66) |
|  |  | ZEN2 | 0.04 (0.03 \| 0.09) | 1.23 (0.70 \| 2.24) |
|  | p-value |  | 0.6465 | 0.3528 |

Table S3: Parameters of energy metabolism of sows (LSmeans ± SE) at day -3 (d-3) before farrowing, day 18 ± 1 day (d18) p.p*.* an day 40 (d40) after insemination fed the same gestation/lactation diet at d-3 and d18 in lactation 2 and fed lactation/gestation diet with different levels of Zearalenone (targeted concentration CON = 0 ZEN/kg feed, ZEN1 = 250 ZEN/kg feed, ZEN 2 = 500 µg ZEN/kg feed) in lactation 1 and gestation. Different superscripts indicate significant differences.

|  |  |  | Glucose [mmol/L] | Triglycerides [mmol/L] | Cholesterol [mmol/L] | Non-esterified fatty acids [µmol/L] |
| --- | --- | --- | --- | --- | --- | --- |
| Lactation 1 | d-3 | CON | 3.75 ± 0.09^a^ | 0.64 ± 0.08 | 1.79 ± 0.05 | 333.36 ± 44.01 |
|  |  | ZEN1 | 3.83 ± 0.09^ab^ | 0.61 ± 0.08 | 1.77 ± 0.05 | 436.95 ± 45.00 |
|  |  | ZEN2 | 4.21 ± 0.09^b^ | 0.72 ± 0.08 | 1.80 ± 0.05 | 255.96 ± 44.94 |
|  | d18 | CON | 5.17 ± 0.09^c^ | 0.22 ± 0.08 | 1.68 ± 0.06 | 267.54 ± 45.56 |
|  |  | ZEN1 | 5.13 ± 0.09^c^ | 0.18 ± 0.08 | 1.62 ± 0.06 | 185.46 ± 45.64 |
|  |  | ZEN2 | 5.18 ± 0.09^c^ | 0.23 ± 0.08 | 1.61 ± 0.06 | 261.38 ± 45.75 |
| Gestation | d40 | CON | 3.59 ± 0.09^a^ | 0.63 ± 0.08 | 1.64 ± 0.06 | 101.00 ± 45.55 |
|  |  | ZEN1 | 3.59 ± 0.09^a^ | 0.66 ± 0.08 | 1.71 ± 0.06 | 80.81 ± 45.81 |
|  |  | ZEN2 | 3.43 ± 0.09^a^ | 0.42 ± 0.08 | 1.77 ± 0.06 | 112.33 ± 46.56 |
| Lactation 2 | d18 | CON | 4.75 ± 0.09^e^ | 0.45 ± 0.08 | 2.05 ± 0.06 | 417.01 ± 45.55 |
|  |  | ZEN1 | 4.63 ± 0.09^e^ | 0.41 ± 0.08 | 2.03 ± 0.06 | 368.65 ± 45.82 |
|  |  | ZEN2 | 4.77 ± 0.10^e^ | 0.34 ± 0.08 | 2.09 ± 0.06 | 446.59 ± 48.42 |
| p-values | | | | | | |
|  |  | day | **<0.0001** | **<0.0001** | **<0.0001** | **<0.0001** |
|  |  | group | 0.1987 | 0.5480 | 0.6299 | 0.9213 |
|  |  | group*day | **0.0219** | 0.3158 | 0.7556 | 0.0714 |

Table S4: Parameters of electrolyte metabolism of sows (LSmeans ± SE) at day -3 (d-3) before farrowing, day 18 ± 1 day (d18) p.p*.* an day 40 (d40) after insemination fed the same gestation/lactation diet at d-3 and d18 in lactation 2 and fed lactation/gestation diet with different levels of Zearalenone (targeted concentration CON = 0 ZEN/kg feed, ZEN1 = 250 ZEN/kg feed, ZEN 2 = 500 µg ZEN/kg feed) in lactation 1 and gestation. Different superscripts indicate significant differences.

|  |  |  | Chloride [mmol/L] | Sodium [mmol/L] | Potassium [mmol/L] | Calcium [mmol/L] | Phosphorus [mmol/L] |
| --- | --- | --- | --- | --- | --- | --- | --- |
| Lactation 1 | d-3 | CON | 114.90 ± 0.70 | 148.56 ± 0.74 | 5.29 ± 0.07 | 2.42 ± 0.02 | 2.30 ± 0.04 |
|  |  | ZEN1 | 115.08 ± 0.72 | 148.53 ± 0.77 | 5.29 ± 0.08 | 2.41 ± 0.02 | 2.27 ± 0.04 |
|  |  | ZEN2 | 113.78 ± 0.73 | 147.66 ± 0.77 | 5.22 ± 0.08 | 2.44 ± 0.02 | 2.34 ± 0.04 |
|  | d18 | CON | 113.90 ± 0.75 | 149.45 ± 0.80 | 4.81 ± 0.08 | 2.73 ± 0.02 | 1.50 ± 0.04 |
|  |  | ZEN1 | 113.05 ± 0.83 | 149.15 ± 0.89 | 4.89 ± 0.09 | 2.75 ± 0.02 | 1.50 ± 0.04 |
|  |  | ZEN2 | 112.99 ± 0.80 | 148.80 ± 0.87 | 5.05 ± 0.08 | 2.69 ± 0.02 | 1.44 ± 0.04 |
| Gestation | d40 | CON | 105.09 ± 0.72 | 142.04 ± 0.77 | 4.87 ± 0.08 | 2.60 ± 0.02 | 2.02 ± 0.04 |
|  |  | ZEN1 | 105.01 ± 0.73 | 141.91 ± 0.78 | 4.94 ± 0.08 | 2.64 ± 0.02 | 2.05 ± 0.04 |
|  |  | ZEN2 | 105.29 ± 0.76 | 142.09 ± 0.80 | 4.89 ± 0.08 | 2.61 ± 0.03 | 1.98 ± 0.04 |
| Lactation 2 | d18 | CON | 107.77 ± 0.72 | 144.86 ± 0.77 | 4.59 ± 0.08 | 2.59 ± 0.02 | 1.74 ± 0.04 |
|  |  | ZEN1 | 107.00 ± 0.73 | 144.33 ± 0.78 | 4.65 ± 0.08 | 2.62 ± 0.02 | 1.70 ± 0.04 |
|  |  | ZEN2 | 104.65 ± 0.78 | 142.78 ± 0.83 | 4.55 ± 0.08 | 2.58 ± 0.03 | 1.75 ± 0.05 |
| p-values | | | | | | | |
|  |  | day | **<0.0001** | **<0.0001** | **<0.0001** | **<0.0001** | **<0.0001** |
|  |  | group | 0.0716 | 0.2847 | 0.6288 | 0.3673 | 0.9074 |
|  |  | group*day | 0.3851 | 0.9045 | 0.4746 | 0.6393 | 0.6273 |
